# Utilization of whole exome sequencing to identify hereditary mutations in Palestinian families with hereditary cancers

**DOI:** 10.3389/fgene.2026.1779001

**Published:** 2026-06-18

**Authors:** Nouar Qutob, Musa Hindiyeh, Fouad Zahdeh, Akram Alian, Rua Thawabteh, Husam Sallam, Abdallah Natsheh

**Affiliations:** 1 Faculty of Graduate Studies, Arab American University, Ramallah, Palestine; 2 Augusta Victoria Hospital, Jérusalem, Israel; 3 Department of Biochemistry, University of Utah School of Medicine, Salt Lake City, UT, United States

**Keywords:** breast cancer, hereditary cancer, mutations, Palestine, whole exome sequencing

## Abstract

**Introduction:**

Hereditary cancers arise from germline pathogenic variants that confer increased lifetime cancer risk. In Palestine, the genetic basis of hereditary cancer predisposition remains limited. The objective of this study is to investigate germline variants associated with hereditary cancer susceptibility in Palestinian families with strong cancer history.

**Methods:**

Families with suspected hereditary breast cancer were recruited, and germline DNA extracted from peripheral blood was first analyzed using a BRCA1/2 panel. Whole-exome sequencing (WES) was subsequently performed in BRCA-negative probands to identify additional candidate hereditary variants. A total of 34 individuals from three unrelated families were included in the study, comprising 8 affected and 26 unaffected individuals. Segregation analysis and in silico functional assessment were conducted to evaluate variant pathogenicity.

**Results:**

We identified a splice‐site variant in RAD50 (c.2524+3A>G) in Family I, a truncating MSH4 variant (c.328C>T; p.R110X) and a missense STAT6 variant (c.1216C>G; p.L406V) in Family II, and a splice‐region PRKAR1A variant (c.973+6T>C) in Family III. These variants are segregated with disease in the respective families and are predicted to affect protein function.

**Conclusion:**

Our findings highlight the importance of germline genomic analysis in characterizing hereditary cancer predisposition in underrepresented populations and provide preliminary data for future risk assessment and genetic counseling strategies in Palestine.

## 1 Introduction

Cancer development involves the accumulation of genetic alterations, which can be classified as either somatic or gernline. While most cancers arise from acquired somatic mutations (Pleasance et al., 2010; Vogelstein et al., 2013), approximately 5%–10% are attributed to inherited germline variants that confer hereditary cancer predisposition. These germline mutations are present in all cells and may significantly increase lifetime cancer risk within affected families.

Whole-exome sequencing (WES) has emerged as a powerful tool for identifying germline pathogenic variants in families with suspected hereditary cancer syndromes (Pleasance et al., 2010). Unlike tumor sequencing, which focuses on somatic alterations for treatment selection, germline sequencing aims to identify inherited variants relevant to cancer risk assessment, early detection, and genetic counseling. WES is a powerful method that has been widely used for diagnosis of difficult-to-diagnose patients (Iglesias et al., 2014), prenatal diagnosis (Iglesias et al., 2014; Xu et al., 2014), and early diagnosis of debilitating diseases (Xu et al., 2014; Bras and Singleton, 2011). It has proven successful in finding causative mutations that enable accurate and confirmed diagnoses essential for targeted therapies (Iglesias et al., 2014; Sassi et al., 2014; Grossmann et al., 2011; Taneri et al., 2012; Rabbani et al., 2012).

Large-scale international sequencing efforts have substantially expanded our understanding of cancer genetics. Large-scale sequencing analyses have identified numerous recurrently mutated genes and chromosomal translocations, including those affecting the WNT, RAS-MAPK, PI3K, TGF-β, P53, and DNA mismatch-repair pathways (Wood et al., 2007; Sjöblom et al., 2007). For example, The Cancer Genome Atlas (TCGA) consortium has gathered data on over thirty tumor types from more than 11,000 patients, generating multiple data types for each sample including DNA sequence, gene expression, microRNA expression, DNA methylation, protein expression, and chromosomal copy number profiles (Wood et al., 2007). These analyses have substantially expanded knowledge on the mechanisms of tumorigenesis across cancer types. An increasing number of studies are reporting the testing of multiple cancer-associated genes by next-generation sequencing (NGS) (Muzny et al., 2012; Ishaque et al., 2018; Kim et al., 2013; Yurgelun 2015; Fleming et al., 2012).

Therapies directed against specific mutations have demonstrated remarkable clinical efficacy. The FDA-approved BRAF inhibitor vemurafenib, for example, has achieved significant clinical responses in patients harboring specific BRAF mutations (Chapman et al., 2011; Flaherty et al., 2010; Davies et al., 2002; Flaherty et al., 2012). These successes underscore the importance of comprehensive mutation profiling for precision oncology.

Despite these advances globally, genetic disease research in Palestine has focused primarily on treatment mechanisms, with limited attention given to understanding the genetic landscape at the population level (Qumseya et al., 2014). Moreover, existing studies have primarily investigated individual genes or somatic tumor characteristics, while comprehensive germline analyses in high-risk families remain scarce. Palestine presents a fertile ground for genomics research due to its large number of recorded and varied cancer cases; however, systematic interrogation of the genetic landscape has been limited, and multidisciplinary research in this field remains underdeveloped (Qumseya et al., 2014; Hamameh et al., 2017; Ghannam 2018; Ayesh et al., 2018). Analyzing germline genetic variation can enhance understanding of inherited disease risk and inform prevention and personalized healthcare strategies in the Palestinian population. Therefore, advancing genomic research in Palestine through WES represents a critical step toward filling this gap and building the foundation for precision oncology in the region.

This study aimed to investigate germline variants associated with hereditary cancer susceptibility in three Palestinian families who tested negative for BRCA1/2 mutations, using whole-exome sequencing to systematically interrogate the mutational background of probands and identify pathogenic variants that may confer cancer predisposition.

## 2 Methods

### 2.1 Study design and analytical workflow

**FIGURE 1:**
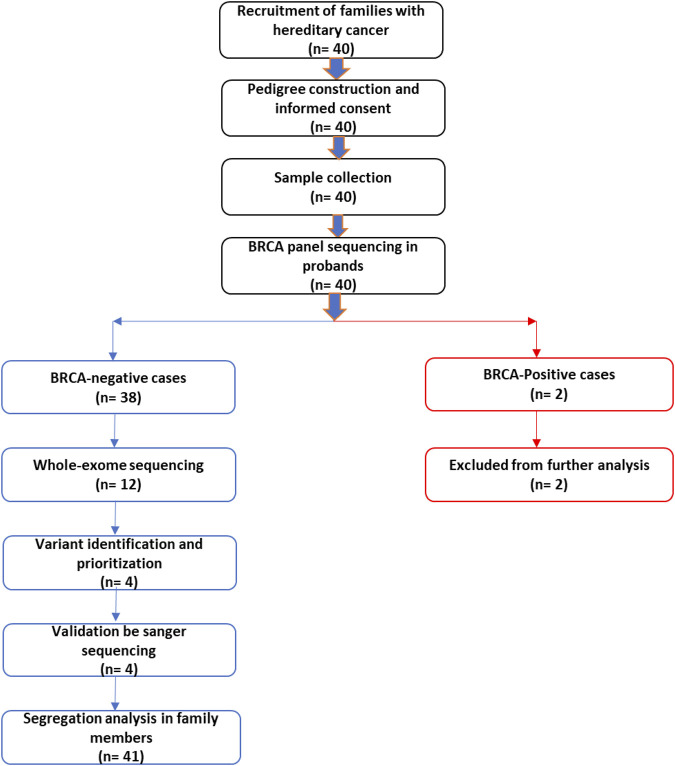
Flowchart of the study design and analysis pipeline. Forty probands were screened using a BRCA panel. BRCA-negative cases (n = 38) were considered for whole-exome sequencing, of which 12 were selected. Variants were prioritized (n = 4) and validated by Sanger sequencing (n = 4). Segregation analysis was performed in family members (n = 41). BRCA-positive cases (n = 2) were excluded from further analysis.

A schematic overview of the study design and analytical pipeline is provided in Figure 1. The flowchart summarizes the overall workflow, including families’ recruitment, BRCA Panel Sequencing, Whole-exome sequencing, WES variant prioritization, and Sanger Sequencing. This visual representation is intended to enhance clarity and facilitate understanding of the stepwise analytical approach.

### 2.2 Families recruitment and sample collection

**FIGURE 2:**
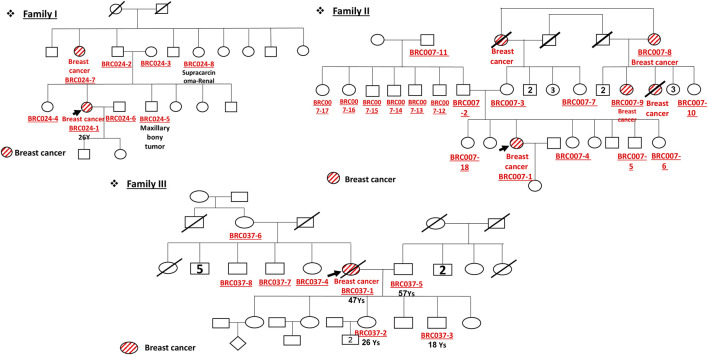
Families’ Pedigrees. The pedigrees structure of three Arab Palestinian families that affected with breast cancer.

Families with a history of hereditary cancers were recruited, and family pedigrees were constructed to guide participant selection. Families with a history of hereditary cancers were recruited for this study. To facilitate the identification of rare germline variants, DNA samples from three families that previously tested negative for *BRCA* mutations were included (Figure 2). In total, 34 individuals from three unrelated families (Families I, II, and III) were analyzed. Family I comprised 8 members, including 4 affected individuals (two diagnosed with breast cancer and two with other cancer types) and 4 unaffected individuals. Family II included 18 members, of whom 3 were affected with breast cancer and 15 were unaffected. Family III consisted of 8 members, including 1 affected individual with breast cancer and 7 unaffected individuals. All patients provided informed consent prior to participation, and the study was approved by the Helsinki Committee for Ethical Approval, Palestinian Health Research Council, Gaza, Palestine (Approval No. PHRC/HC/588/19; renewal application submitted). In addition, the study was approved by the ethical board of Augusta Victoria Hospital. From each proband and selected family members, 3 mL of whole blood was collected in EDTA tubes.

### 2.3 BRCA panel sequencing

Genomic nucleic acid was extracted from peripheral blood sample using the magLEAD ® 12gC (PSS, Japan), DNA was subjected to targeted amplification of specific genomic regions using AmpliSeq for Illumina BRCA Panel, these targets include all exonic regions and flanking intronic sequences of *BRCA1* and *BRCA2* genes. Followed by sequencing on an Illumina iSeq100 instrument. All reportable regions are sequenced at a minimum 500X coverage, unless otherwise mentioned. Variants classified as benign or likely benign are not validated or reported, but are available upon request. DNA variants are classified following ACMG/AMP’s Standards and Guidelines for the Interpretation of Sequence Variants (Richards et al., 2015) using Varsome Clinical Software (Kopanos et al., 2019). The assay was validated using internal and external controls, demonstrating robust performance and suitability for routine clinical testing.

### 2.4 Whole-exome sequencing

DNA was extracted using the Promega Blood kit, catalog #A1120 according to the manufacturer’s instructions. DNA concentrations and purity were assessed using the Thermo Scientific NanoDrop 2000c. WES was performed on the DNA of the patient and the two unaffected siblings, one of them previously diagnosed with a brain tumor. DNA libraries were generated using the Illumina DNA Prep with Enrichment—(S) Tagmentation, 16 Samples kit, catalog # 20025523, following the manufacturer’s instructions. Library yield was determined using the Qubit dsDNA HS Assay Kit, catalog #Q32850, and the mean fragment size was determined using an Agilent Technology 2100 Bioanalyzer with a High-Sensitivity DNA kit, catalog #5067-4626. Denaturation and final loading concentration of the libraries were performed according to the Denature and Dilute Libraries Guide for NextSeq 500 and NextSeq 550 Sequencing Systems. The prepared libraries were sequenced on a NextSeq 550 platform with 40 X depth.

### 2.5 WES variant prioritization

A bioinformatics pipeline was developed to evaluate sequence data from the panels. Following demultiplexing, Sequence data reads (i.e., FASTQ files) were aligned to the reference human genome (hg19) using the Burrows–Wheeler Aligner (v0.7.12). PCR duplicates were removed using SAMtools v0.1.18. Indels were realigned and base quality score was recalibrated with the Genome Analysis Tool Kit (GATK v3.0-0-g6bad1c6) using the recommended parameters. Genotypes were called and filtered using GATK HaplotypeCallerand Variant Filtration tools. Variants were annotated with respect to genomic position, genomic location, and predicted function using *in silico* tools. Variant allele frequencies were noted from gnomAD. Variants were interpreted using the guidelines of the American College of Medical Genetics and Genomics. Mutations were interpreted as potentially damaging if they were truncating, predicted by *in silico* tools to be splice-altering, or were missense mutations with a predicted functional effect.

### 2.6 Sanger sequencing

Variants identified as pathogenic or potentially pathogenic, particularly those with predicted effects on protein function, were prioritized for further study. Finally, segregation analysis was performed using Sanger sequencing of gDNA from family members to validate candidate mutations. Also, RT-PCR followed by Sanger sequencing was performed to investigate the effect of splice site variants at cDNA level.

Polymerase chain reaction (PCR) was utilized to amplify the targeted regions using the following primers in Table 1. Primers were designed using Primer 3 software (https://primer3.ut.ee/). After purification of the PCR products, Sanger sequencing was run using the BigDye Terminator v3 kit (Applied Biosystem) according to the manufacturer’s instructions. The sequences of the PCR products were viewed and analyzed against the human reference genome hg19 (UCSC Genome Browser).

**TABLE 1:** The sequences of the variant’s primers.

Gene name	Primer sequence	
	Forward	Reverse
RAD50	GCA​CAA​CAA​GCA​GCT​AAG​C	GGA​ACT​TCG​TTG​TCC​ATG​TAA​CA
RAD50-cDNA	AAA​AGG​AAA​AGC​GGC​GTG​AT	TCT​CGC​ATT​CAC​TTA​GTT​GAG​C
RAD50-cDNA inner	CAG​CGC​CTA​AAG​AAC​GAC​AT	CCT​GCT​GGT​CCT​GTA​TAA​GC
MSH4	TGG​AAA​CAA​AAG​AGC​TTA​TGC​AGA	ACA​TCA​TCT​AGG​TTA​ATC​TTC​CAC​T
STAT6	CCC​ATG​TTA​GAA​CCC​ACC​CT	CCT​GTC​CTC​ACC​CTC​TTC​AG
PRKAR1A	TGC​TAC​AAC​GTC​GGT​CAG​AA	CCT​TCC​CTC​TCA​GAG​CCA​AA

## 3 Results

### 3.1 WES analysis identified variants in genes for cancer germline predisposition

In order to identify germline variants predisposing to breast cancer familial cancers, we performed BRCA Panel sequencing to 39 high risk cancer patients. Of the 39 patients, 2 (5.1%) patients had a mutated BRCA2 gene (one patient with c.9382C>T and the other patient with c.2254_2257del + c.5351dup. In order to identify rare germline variants, we performed WES on DNA samples that tested negative for BRCA in three families, as shown in (Figure 2) (Abramson et al., 2024).

WES data is available at the NCBI database under the BioProject submission: SUB16049434.

#### 3.1.1 Family (I)

Family I was selected for WES due to early-onset breast cancer in the proband and additional tumors in paternal relatives. A rare RAD50 splice-region variant was identified and further assessed for its possible role in hereditary cancer predisposition.

### 3.2 Clinical overview of family I

Family I included a patient diagnosed with right breast invasive ductal carcinoma, staged as T1N1M0, indicating tumor involvement with metastasis to 1–3 regional lymph nodes and no evidence of distant metastasis. Immunohistochemical analysis showed that the tumor was estrogen receptor (ER) positive and HER2 positive (3+). The patient received neoadjuvant chemotherapy consisting of doxorubicin and cyclophosphamide (AC) followed by paclitaxel. Subsequently, she underwent modified radical mastectomy (MRM) with axillary lymph node dissection (ALND), followed by breast reconstruction. Postoperatively, the patient received radiotherapy and continued her oncology treatment at another medical center.

Based on the available pedigree information and reported clinical history, the brother was diagnosed with Maxillary bony tumor, the paternal Aunt was diagnosed with breast Cancer and the paternal aunt with Supra renal carcinoma. The clustering of breast cancer cases and vertical transmission pattern supported further germline investigation for hereditary cancer predisposition (Table 2).

**TABLE 2:** Clinicopathological characteristics and segregation analysis of the three studied families.

Family	ID	Relationship	Age	Affected status	Cancer type/Clinical phenotype and age of diagnosis	Variant status	Genotype	Gene/Inheritance pattern	Variant
I	BRC024-1	Proband	27	Affected	Right breast, invasive ductal carcinoma, ER positive, Her2 positive (+3). Cancer present in 1-3 neighboring lymph nodes with no spread to distant organs, diagnosed in 10/2022	Carrier	AG	RAD50/Autosomal dominant	c.2524+3A>G
I	BRC024-2	Father	62	Unaffected	None	Carrier	AG	RAD50/Autosomal dominant	c.2524+3A>G
I	BRC024-5	Brother	30	Affected	Maxillary bony tumor	Carrier	AG	RAD50/Autosomal dominant	c.2524+3A>G
I	BRC024-3	Mother	50	Unaffected	None	Wild-type	AA	—	—
I	BRC024-4	Sister	—	Unaffected	None	Wild-type	AA	—	—
I	BRC024-6	Husband	30	Unaffected	None	Wild-type	AA	—	—
I	BRC024-7	Paternal aunt	—	Affected	Breast cancer diagnosed at age 50	Wild-type	AA	—	—
I	BRC024-8	Paternal aunt	—	Affected	Suprarenal carcinoma	Wild-type	AA	—	—
II	BRC007-1 (MSH4, c.328C>T (p.R110*))	Proband	30	Affected	Invasive ductal carcinoma with mucinous features, grade 2-3. ER positive HER-2 negative. Metastatic from initial diagnosis (bone and liver metastases), diagnosed in 5/2023	Carrier	CT	MSH4/Autosomal recessive	c.328C>T (p.R110*)
II	BRC007-2	Father	65	Unaffected	None	Carrier	CT	MSH4/Autosomal recessive	c.328C>T
II	BRC007-4	Sister	—	Unaffected	None	Carrier	CT	MSH4/Autosomal recessive	c.328C>T
II	BRC007-(12 + 13+14 + 15)	Paternal cousins (n = 4)	—	Unaffected	None	Carrier	CT	MSH4/Autosomal recessive	c.328C>T
II	BRC007-(16 + 17)	Paternal aunts (n = 2)	—	Unaffected	None	Carrier	CT	MSH4/Autosomal recessive	c.328C>T
II	BRC007-3	Mother	52	Unaffected	None	Wild-type	CC	—	—
II	BRC007-5	Brother	—	Unaffected	None	Wild-type	CC	—	—
II	BRC007-(6 + 18)	Sisters (n = 2)	—	Unaffected	None	Wild-type	CC	—	—
II	BRC007-7	Maternal aunt	—	Unaffected	None	Wild-type	CC	—	—
II	BRC007-11	Paternal grandmother	—	Unaffected	None	Wild-type	CC	—	—
II	BRC007-1 (STAT6, c.1216C>G (p.L406V))	Proband	30	Affected	Invasive ductal carcinoma with mucinous features, grade 2-3. ER positive HER-2 negative. Metastatic from initial diagnosis (bone and liver metastases), diagnosed in 5/2023	Carrier	GC	STAT6/Autosomal dominant	c.1216C>G (p.L406V)
II	BRC007-3	Mother	52	Unaffected	None	Carrier	GC	STAT6/Autosomal dominant	c.1216C>G
II	BRC007-(4 + 6)	Sisters (2)	—	Unaffected	None	Carrier	GC	STAT6/Autosomal dominant	c.1216C>G
II	BRC007-5	Brother	—	Unaffected	None	Carrier	GC	STAT6/Autosomal dominant	c.1216C>G
II	BRC00-8	Mom’s maternal aunt	—	Affected	Breast cancer	Carrier	GC	STAT6/Autosomal dominant	c.1216C>G
II	BRC007-9	Mom’s maternal cousin	—	Affected	Breast cancer	Carrier	GC	STAT6/Autosomal dominant	c.1216C>G
II	BRC007-2	Father	—	Unaffected	None	Wild-type	GG	​	​
II	BRC007-7	Maternal aunt	​	Unaffected	None	Wild-type	GG	​	​
II	BRC007-10	Mom’s maternal cousin	​	Unaffected	None	Wild-type	GG	​	​
III	BRC037-1	Proband	48	Affected	Bilateral breast cancer, underwent bilateral lumpectomy, right breast tissue showed multifocal invasive ductal carcinoma grade 3. The left breast showed invasive ductal carcinoma grade 3. Tissue was triple negative (ER, PR and HER-2) KI-67 30%	Carrier	TC	PRKAR1A/Autosomal dominant	c.973+6T>C
III	BRC037-2	Daughter	—	Unaffected	None	Carrier	TC	PRKAR1A/Autosomal dominant	c.973+6T>C
III	BRC037-6	Mother	—	Unaffected	None	Carrier	TC	PRKAR1A/Autosomal dominant	c.973+6T>C
III	BRC037-3	Son	—	Unaffected	None	Wild-type	TT	—	​
III	BRC037-5	Husband	—	Unaffected	None	Wild-type	TT	—	​
III	BRC037-4	Sister	—	Unaffected	None	Wild-type	TT	—	​
III	BRC037-(7 + 8)	Brothers (2)	—	Unaffected	None	Wild-type	TT	—	​

### 3.3 RAD50 and DNA repair dysfunction

Our genetic analysis identified a variant in the RAD50 gene, which is located on chromosome 5 and inherited in an autosomal dominant manner. The detected variant lies within a splicing region, where adenine (A) is replaced by guanine (G) at position c.2524+3 (c.2524+3A>G) with a donor loss score of 0.01 and Donor Gain score or 0.03.

**FIGURE 3:**
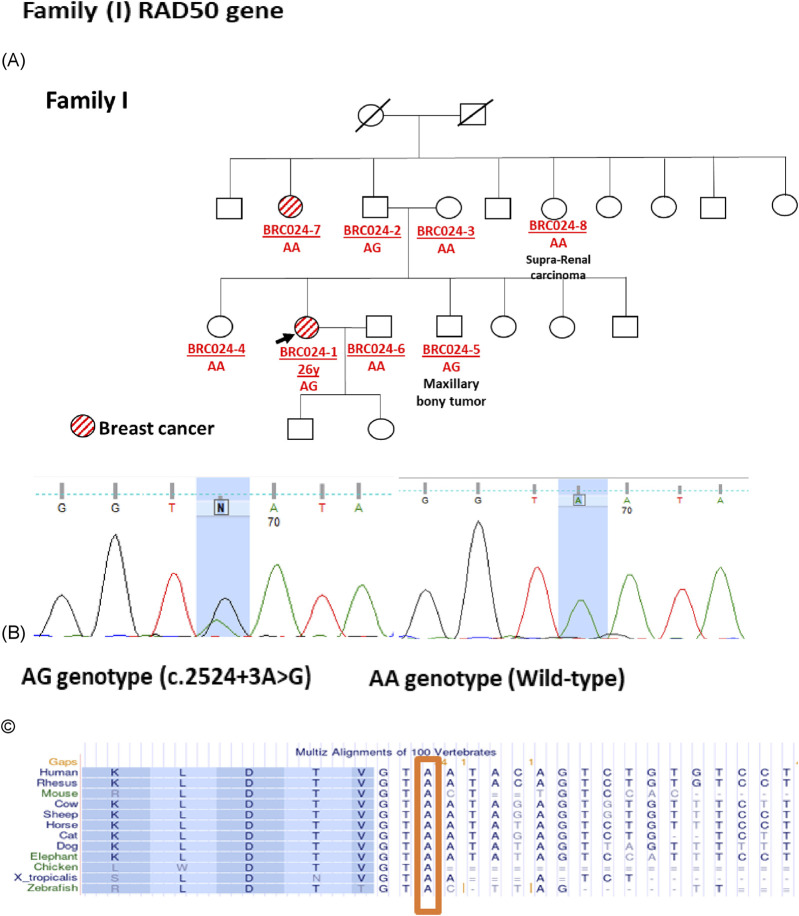
Family segregation of RAD50 Variant. **(A)** Genotype results of each family member shown in the family pedigree. **(B)** Sanger sequencing results of the detected variant in Family I. **(C)** Conservation analysis of RAD50 c.2524+3A locus using UCSC Genome browser.

Segregation analysis was conducted to evaluate the distribution of the variant within the family. The proband, a 27-year-old female diagnosed with invasive ductal carcinoma (IDC), grade III, was identified as a heterozygous carrier of the variant (AG). Her father, a 62- year-old unaffected male, and her brother, a 26-year-old male diagnosed with a maxillary bony tumor, were also heterozygous carriers. In contrast, several family members were found to be wild-type (AA), including the proband’s mother (60 years old, unaffected), sister (unaffected), and husband (30 years old, unaffected). Two paternal aunts were also tested and were wild-type: one who had previously been diagnosed with breast cancer at the age of 50 and recovered following treatment, and another diagnosed with suprarenal carcinoma. Overall, the variant was detected in the proband and her brother, both of whom are affected by tumors, as well as in the unaffected father, while it was absent in other tested relatives, including individuals affected by different cancer types. The familial segregation pattern is illustrated in Figures 3A,B.

**FIGURE 4:**
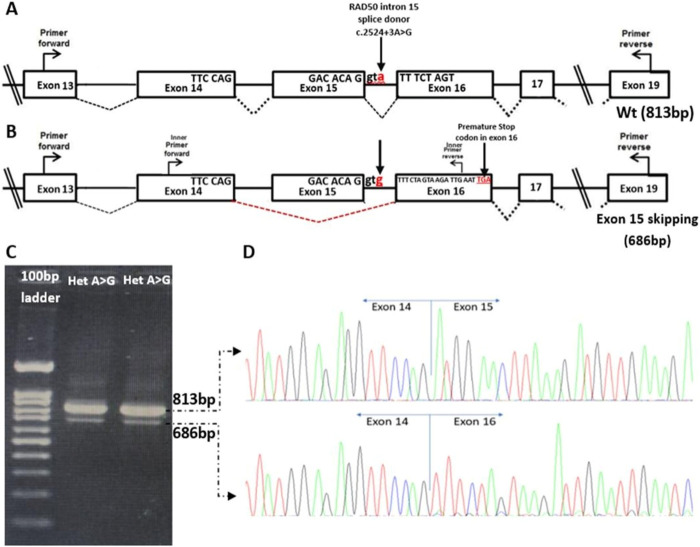
cDNA analysis of the mutated RAD50: c.2524+3A>G donor splice site. **(A)** Schematic representation of the exon/intron structure of the RAD50 gene up and downstream of the mutated 2524+3A>G splice site in intron 15 (arrow). Primers used for amplification are indicated (as shown in Table 1). **(B)** Schematic representation of the aberrant splice product observed in individuals carrying the heterozygous RAD50: c.2524+3A>G splicing mutation (arrow). cDNA analysis revealed an abnormal splice variant in which exon 14 is directly joined to exon 16, resulting in exon 15 skipping, and leading to a frameshift and the introduction of a premature termination codon (TGA) at the beginning of exon 16. **(C)** 2% Agarose gel electrophoresis of RAD50 cDNA analysis resulted in 813bp fragment for wild-type allele, and an additional fainter and smaller fragment for mutated allele, which corresponds to exon 15 skipping, is only present in the carrier samples. 100 bp Ladder was used **(D)** Chromatograms of sanger sequencing results of the PCR fragments. Sequencing results of the small fragment demonstrate that exon 14 is spliced directly to exon 16 indicating that the RAD50: c.2524+3C>A mutation may alter the strength of the intron 15 splice donor site.

**FIGURE 5:**
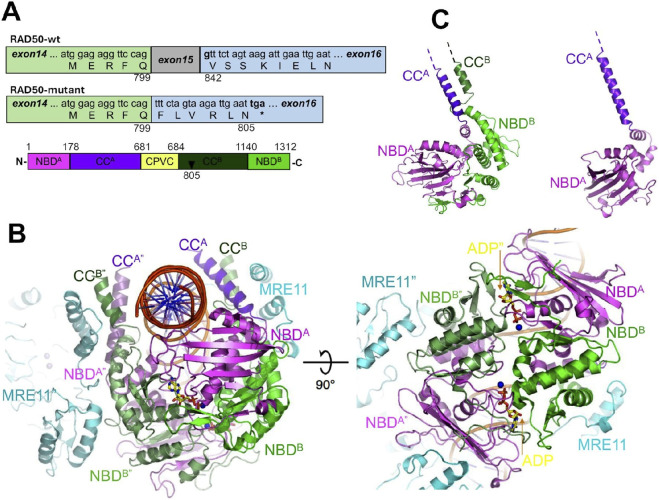
Structural organization of Rad50. **(A)** Organization of RAD50 wild type (wt) and mutant genes around exons 14 (light green), 15 (grey) and 16 (light blue). Single letter amino acid translation are shown below the genetic codes. Amino acid numbering are shown below the boxes. A guanine nucleotide (bold letter ‘g’ in wt) is lost upon skipping of exon-15 and fusion to exon-16 resulting in frame shift in the mutant and a premature termination (*) of protein after residue 805. Motifs of RAD50 protein are shown depicting the nucleotide binding domain (NBD) in magenta for the N-terminal (N-) domain (NBD^A^) and green for the C-terminal (-C) domain (NBD^B^). Coiled coil (CC) domains are shown in purple and denoted CC^A^ and CC^B^ for the N- and C-terminal domains, respectively. CPVC (yellow) shows the Zn2+ binding hook. Amino acid boundaries for each motif are shown. A black triangle at 805 depicts the truncation point of the mutant protein. **(B)** Structure of a pair of Mre11-RAD50 heterodimer homolog in the cutting state (PDB: 6S85). Color code as in **(A)**. Quotation marks (”) depict the second protomer of each dimer. One unit of RAD50 NBD^A^/NBD^B^ pair forms a dimer with another NBD^A^/NBD^B^ pair to bind Mre11 dimer. Coiled coils (CC) of RAD50 dimer clamp around DNA (orange/blue double helix) forming DNA binding template (left). ADP is shown in yellow sticks and Mg^2+^ in blue spheres. A 90° twist view showing the dimer interface of the two RAD50 NBD^A^/NBD^B^ pairs creating the ADP binding pocket. ATP binding stabilizes the NBD assembly and creates the DNA-binding platform. **(C)** Structures of NBD^A^ (magenta) and NBD^B^ (green) of wt RAD50 dimerizing (left; PDB: 3QG5) and of mutant (right, only NBD^A^, predicted using AlphaFold (Abramson et al., 2024). Dashed lines depict extensions of the very long coiled coils (CC). Structural images made with PyMol (Schrödinger, LLC.).

To assess whether the RAD50 c.2524+3A locus is evolutionarily conserved, we analyzed it using the UCSC Genome Browser. The results indicated that RAD50 c.2524+3A lies within a highly conserved genomic region (Figure 3C). To further support the hypothesis that the RAD50 (c.2524+3A>G) variant is the causative mutation in the tested family, we performed cDNA analysis on samples from carrier family members. The results confirmed exon 15 skipping, as shown in (Figure 4). In silico analysis revealed a fusion between exon 14 and exon 16 resulting in a frameshift and the creation of a premature termination codon (TGA) at the start of exon 16 and the production of a truncated 805 amino acid protein. Using the dbscSNV database, the RAD50 c.2524+3A>G variant was predicted to be deleterious with a score of 0.9994. In contrast, SpliceAI predicted a low impact on splicing (score = 0.03). Structural analysis further showed that the truncated mutant lacks the C-terminal coiled coils (CC) and nucleotide binding domain (NBD) (Figure 5).

#### 3.3.1 Family (II)

Family II was analyzed because of young-onset metastatic breast cancer and a family history of breast cancer. WES identified variants in SLC22A18, MSH4, and STAT6, which were evaluated by Sanger sequencing and segregation analysis.

### 3.4 Clinical overview of family II

Family II included a patient who was diagnosed with invasive ductal carcinoma with mucinous features, grade 2–3, which was estrogen receptor (ER) positive and HER2 negative. The disease was metastatic at the time of initial diagnosis. The patient received palliative radiotherapy and was started on a CDK4/6 inhibitor as part of first-line systemic therapy. Following disease progression, treatment was switched to exemestane (Aromasin) in combination with everolimus. Subsequent therapy included goserelin (Zoladex) and zoledronic acid (Zometa). Due to further disease progression, the patient was later started on paclitaxel in combination with zoledronic acid.

Based on the available pedigree information and reported clinical history, the mother’s paternal aunt was diagnosed with Breast Cancer, and the mother’s maternal cousin was diagnosed with Breast Cancer. The pedigree suggested possible hereditary predisposition, although the inheritance pattern appeared less straightforward, potentially reflecting genetic heterogeneity. No consistent non-breast malignancies were documented in the available family history (Table 2).

Our genetic analysis identified three variants: SLC22A18 (c.404-2A>C), MSH4 (c.328C>T, p.R110X) and STAT6 (c.1216C>G, p.L406V). All variants underwent segregation validation by Sanger Sequencing. Only one variant: SLC22A18 (c.404-2A>C) did not segregate within the family. All members of Family II were found to be wild type (AA) for this variant.

### 3.5 MSH4 and meiotic recombination pathways

**FIGURE 6:**
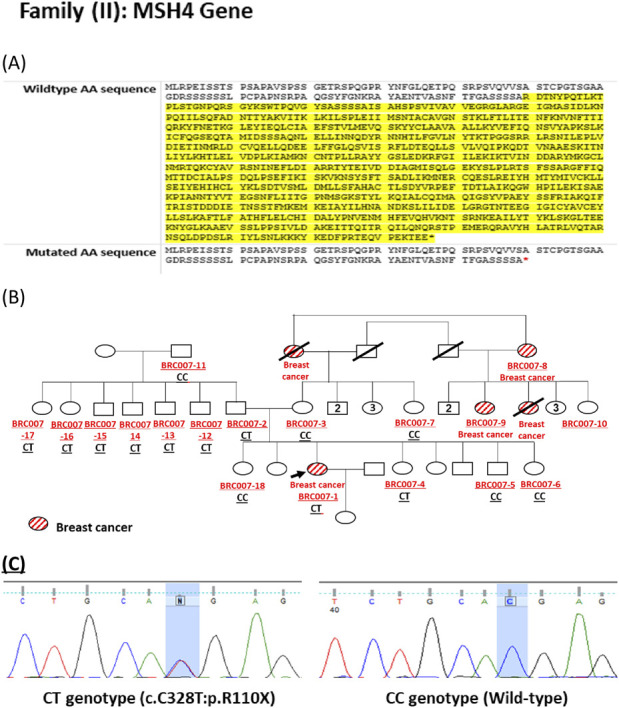
Family segregation of MSH4 Variant. **(A)** Amino acid sequences for the wild-type and mutant MSH4 protein; all amino acids in yellow color are lost. (https://www.mutationtaster.org). **(B)** Genotype results of each family member shown in the family pedigree. **(C)** Sanger sequencing results of the detected variant in Family II.

The second variant identified in Family II was a nonsense mutation in the MSH4 gene (c.328C>T, p.R110*), located on chromosome 1 and inherited in an autosomal recessive manner. This variant results from a cytosine-to-thymine substitution at nucleotide position c.328, generating a premature stop codon at amino acid position 110, compared with the full-length protein of 937 amino acids (Figure 6A). The variant was validated by Sanger sequencing, and segregation analysis was performed in the available members of Family II (Figures 6B,C). The proband, a 30-year-old female diagnosed with estrogen receptor–positive, HER2-negative metastatic breast cancer with bone and liver metastases, was identified as a heterozygous carrier of the variant (CT). Her father (65 years old, unaffected), one sister (unaffected), four paternal cousins, and two paternal aunts (all unaffected) were also heterozygous carriers. In contrast, several other relatives carried the wild-type genotype (CC), including the proband’s mother (52 years old, unaffected), brother (unaffected), two sisters (unaffected), maternal aunt (unaffected), and paternal grandmother (unaffected).

**FIGURE 7:**
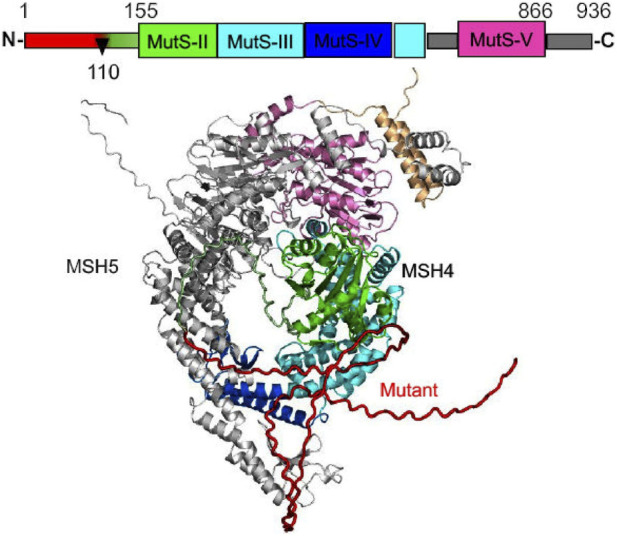
Structural architecture of MSH4-MSH5 heterodimeric model. MSH4 comprises classical MutS Domain-II (green), Domain-III (cyan), Domain-IV (blue), and Domain-V (pink). MSH5 is shown with grey color. The N-terminal fragment (amino acids 1-155) of MSH4 is unstructured (1-109 in red, and 110-155 in pale green). MSH4^R110X^ mutant results in a truncated fragment of 109 residues in length (red) that is predicted to be unstructured. The heterodimer structure was predicted using Alphafold (Abramson et al., 2024). Figure prepared with PyMol (Schrödinger, LLC.).

Comparison of genotype distribution with the clinical status of family members showed that the variant was present in multiple unaffected relatives and therefore did not strictly co-segregate with the disease phenotype. This observation may indicate incomplete penetrance or suggest the contribution of additional genetic or environmental factors to disease development. Structural analysis of this MSH4 c.328C>T (R110X) mutant shows the insertion of a stop codon after Ala109 resulting in a truncated fragment of 109 residues in length, which is predicted to be unstructured (Figure 7).

### 3.6 STAT6 and immune evasion mechanisms

**FIGURE 8:**
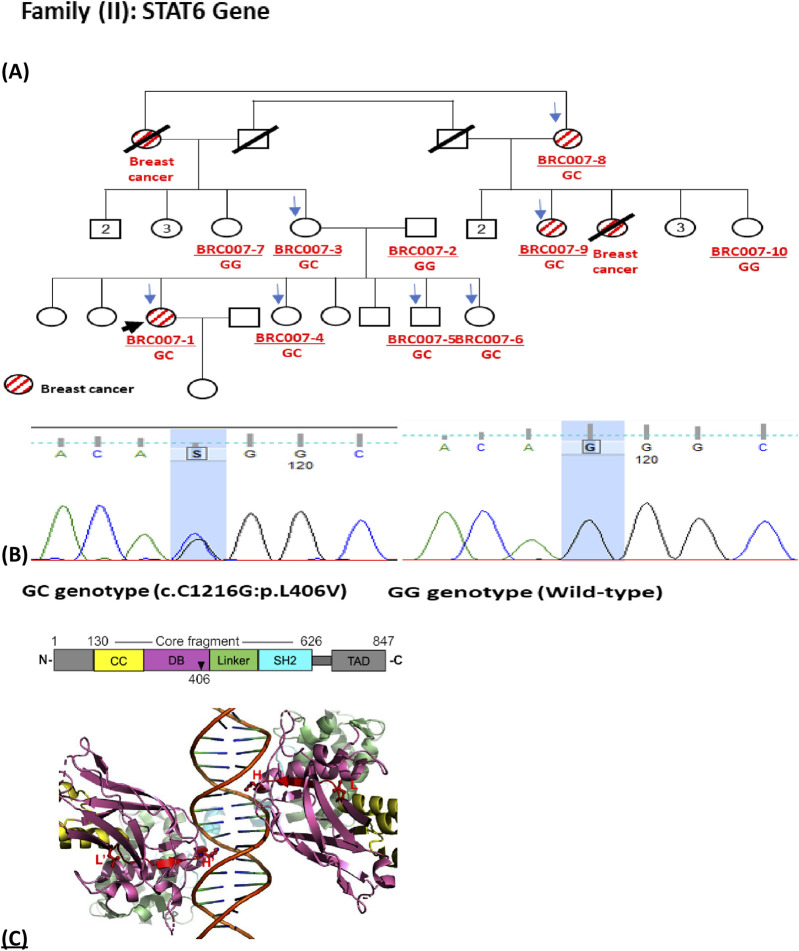
Family segregation of STAT6 Variant. **(A)** Genotype results of each family member shown in the family pedigree. **(B)** Sanger sequencing results of the detected variant in Family II. **(C)** Structure of STAT6 bound to DNA. STAT6 core fragment comprises the coiled coil (CC, yellow), DNA binding (DB, pink), linker (pale green) and SH2 (cyan) domains. A dimer of STAT6 binds DNA with a key residue, His415 (red H & H′ letters), which uniquely identifies N4 position on DNA. Leu406 (red L & L′ letters) is part of the structured DB and extends a few residues to the N-terminus of H415 (red fragment). H415 and L406 are shown in stickballs. Figure prepared with PyMol (Schrödinger, LLC.) Using the crystal structure of STAT6-DNA complex (PDB: 5D39).

The third variant was identified in the STAT6 gene (c.1216C>G, p.L406V), located on chromosome 12, with a REVEL score of 0.586. This missense variant results in a leucine-to-valine substitution at amino acid position 406 and is consistent with an autosomal dominant inheritance pattern. The variant was validated by Sanger sequencing, and the segregation pattern is illustrated in Figures 8A,B.

Segregation analysis revealed that the proband, a 30-year-old female diagnosed with estrogen receptor–positive (ER-positive), HER2-negative metastatic breast cancer with bone and liver metastases, was a heterozygous carrier of the variant (GC). Additional heterozygous carriers included the proband’s mother (52 years old, unaffected), two sisters (unaffected), brother (unaffected), a maternal aunt diagnosed with breast cancer, and a maternal cousin who was also affected by breast cancer.

The remaining tested family members carried the wild-type genotype (CC). The presence of the variant in multiple affected individuals as well as in several unaffected relatives suggests possible incomplete penetrance or age-dependent expression. Notably, two affected relatives from the maternal side of the family were carriers of the variant, which may indicate genetic heterogeneity within this family. Structural analysis showed that residue L406 is located within the DNA-binding (DB) domain of the structured core fragment of STAT6 (Figure 8C) (Heikkinen et al., 2006). This rare missense variant is predicted to be deleterious by several *in silico* prediction tools, including PolyPhen-2, MutationTaster, and SIFT. The STAT6 gene belongs to the signal transducer and activator of transcription (STAT) family of transcription factors, which are known to play important roles in breast cancer tumorigenesis (Lu et al., 2019).

#### 3.6.1 Family (III)

Family III was included due to bilateral triple-negative breast cancer, recurrence, and brain metastasis in the proband. A PRKAR1A splice-region variant was identified in the maternal lineage and evaluated as a potential hereditary risk factor.

### 3.7 Clinical overview of family III

Family III included a patient diagnosed with bilateral breast cancer and underwent bilateral lumpectomy. Histopathological examination revealed multifocal invasive ductal carcinoma, grade 3, in the right breast, while the left breast showed invasive ductal carcinoma, grade 3. Immunohistochemical analysis demonstrated a triple-negative phenotype (ER-, PR-, and HER2-negative) with a Ki-67 proliferation index of 30%. The patient later developed recurrent disease, confirmed by a tru-cut biopsy of the left breast, which revealed recurrent invasive ductal mammary carcinoma, grade 3, maintaining a triple-negative phenotype with an increased Ki-67 index of 80%. Subsequently, the patient developed brain metastases and received radiotherapy to the brain and cervical spine.

Based on the available pedigree information and reported clinical history, the maternal uncle had an unknown type of cancer. The pedigree suggested a possible autosomal-dominant inheritance pattern based on the distribution of affected individuals across generations. The aggressive phenotype and familial clustering warranted germline variant analysis to investigate potential hereditary cancer predisposition factors (Table 2).

### 3.8 PRKAR1A and aberrant PKA signaling

The PRKAR1A variant (c.973+6T>C) was identified in the proband in a heterozygous state and segregated with the maternal side of the family. According to information provided by family members, the proband’s maternal uncle (the mother’s brother) died from cancer, although the specific cancer type remains unknown. Because the variant was inherited from the maternal lineage, it is possible that the maternal uncle may also have carried this variant. However, he was not tested as he is deceased, and his offspring declined both genetic testing for this variant and participation in the study.

**FIGURE 9:**
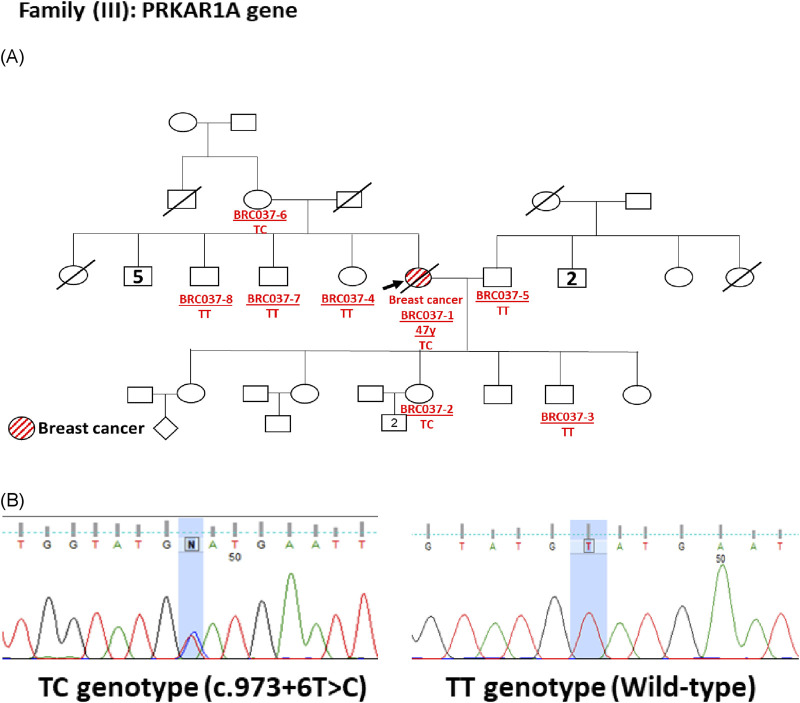
Family segregation of PRKAR1A Variant. **(A)** Genotype results of each family member shown in the family pedigree. **(B)** Sanger sequencing results of the detected variant in Family III.

The variant was validated by Sanger sequencing, and segregation analysis was performed among the available family members (Figure 9). The proband, a 48-year-old female diagnosed with triple-negative invasive ductal carcinoma (IDC), was identified as a heterozygous carrier of the variant (TC). Her daughter (unaffected) and mother (unaffected) were also heterozygous carriers. The remaining tested family members carried the wild-type genotype (TT).

In silico splicing prediction tools yielded inconsistent results regarding the potential impact of this variant on RNA splicing. SpliceAI predicted a low probability of splicing disruption (score = 0.05), whereas the dbscSNV database suggested a high likelihood of deleterious splicing effects (score = 0.9922).

## 4 Discussion

This study represents an initial systematic effort in Palestine to investigate hereditary cancer predisposition using whole-exome sequencing. Our findings identified potentially pathogenic germline variants in DNA repair and signaling pathway genes (RAD50, MSH4, STAT6, and PRKAR1A), emphasizing the value of genomic approaches for uncovering underreported variants in populations that remain underrepresented in global cancer genomics databases.

Among our findings, the RAD50 splice-site variant warrants particular attention in the context of global research. RAD50 splice-site variants and mutations have been previously reported in hereditary breast and ovarian cancer cohorts in European populations and are recognized as cancer susceptibility variants (D'Amours and Jackson, 2002; Stracker and Petrini, 2011; Käshammer et al., 2019; Heikkinen et al., 2006; Lu et al., 2019). In European-ancestry breast cancer families, RAD50 mutations have been identified as contributing to hereditary predisposition, with studies demonstrating that both truncating and splice-site variants can disrupt the MRN complex function (D'Amours and Jackson, 2002; Stracker and Petrini, 2011; Käshammer et al., 2019; Heikkinen et al., 2006). However, RAD50 mutations have been less frequently characterized in populations from the Middle East, North Africa, and South Asia, suggesting either true population-specific variation or insufficient genomic screening in these regions. To our knowledge, systematic characterization of RAD50 variants in Palestinian populations has been limited; thus, this study contributes novel data to understanding cancer predisposition in the Palestinian healthcare context (Hamameh et al., 2017). Beyond breast cancer, RAD50 mutations have been implicated in predisposition to ovarian cancer (D'Amours and Jackson, 2002; Stracker and Petrini, 2011; Heikkinen et al., 2006; Lu et al., 2019), and emerging evidence suggests potential associations with gastric and pancreatic cancers in populations with hereditary cancer syndromes, although these associations remain less characterized than breast and ovarian cancer risk (D'Amours and Jackson, 2002; Stracker and Petrini, 2011; Käshammer et al., 2019). The MRN complex’s central role in DNA double-strand break repair makes RAD50 dysfunction relevant across multiple cancer types where genomic instability is a driving feature (D'Amours and Jackson, 2002; Stracker and Petrini, 2011).

In contrast to the relatively well-characterized RAD50 gene, the MSH4 variant we identified represents a more novel contribution to hereditary cancer genomics. The MSH4 nonsense mutation (c.328C>T, p.R110X) identified in Family II is particularly noteworthy because pathogenic MSH4 variants have rarely been characterized in cancer populations. Most documented MSH4 mutations appear in studies of meiotic recombination and male infertility in populations such as Europeans and East Asians, with limited reports in cancer populations generally (Kneitz et al., 2000; Wang et al., 1999). Emerging evidence suggests that MSH4 may contribute to genomic stability beyond its canonical meiotic role, though this function has not been extensively explored in hereditary cancer cohorts (Cohen et al., 2006). The identification of a pathogenic MSH4 variant in a Palestinian family with hereditary breast cancer represents a potentially novel finding that expands our understanding of this gene’s role in cancer predisposition. MSH4 loss-of-function could theoretically increase susceptibility to multiple cancer types characterized by genomic instability, including colorectal, endometrial, and other mismatch-repair-related malignancies, though direct associations have not been established in the literature (Kneitz et al., 2000; Wang et al., 1999; Cohen et al., 2006).

Similarly, the STAT6 variant identified in Family III demonstrates the complexity of variant interpretation in understudied populations. The STAT6 missense variant (p.L406V) identified in Family III has been previously documented in population databases (dbSNP, 1000 Genomes Project, ActiveDriverDB) (Sherry et al., 2001; 1000 Genomes Project Consortium et al., 2015; Krassowski et al., 2018) and has been identified in cancer-sequencing databases, though its pathogenic significance requires further characterization. STAT6 dysregulation and constitutive activation have been associated with breast cancer metastasis and progression in multiple populations, including European and Asian cohorts (Takeda and Akira, 2012; Ma et al., 2014; Park et al., 2013; Guo et al., 2018). Beyond breast cancer, STAT6 overexpression and activation have been reported in lymphomas, gastric cancer, colorectal cancer, and hepatocellular carcinoma in populations from diverse geographic origins (Takeda and Akira, 2012; Ma et al., 2014; Park et al., 2013; Guo et al., 2018), suggesting that STAT6 represents a broadly relevant oncogenic driver across cancer types. The IL-4/IL-13 signaling pathway upstream of STAT6, which promotes Th2-type immune responses, has been shown to enhance tumor proliferation, epithelial-mesenchymal transition (EMT), and metastatic behavior across multiple cancer models (Takeda and Akira, 2012; Ma et al., 2014; Park et al., 2013; Guo et al., 2018). The specific L406V variant identified in this Palestinian family warrants functional characterization to determine whether this particular substitution confers similar oncogenic properties as other characterized STAT6 mutations.

Finally, the fourth variant we identified—a PRKAR1A splicing mutation—links our findings to well-characterized genetic cancer syndromes. The PRKAR1A splicing variant (c.973+6T>C) identified in Family III connects to a well-established association with Carney complex (CNC) and hereditary malignancy predisposition. PRKAR1A mutations have been identified in patients with Carney complex across diverse populations, including Europeans, North Americans, and Latin Americans (Kirschner et al., 2000; Bertherat et al., 2009; Rothenbuhler and Stratakis, 2010). Women with pathogenic PRKAR1A variants exhibit significantly elevated incidence of breast cancer compared with the general population (Vaduva et al., 2024), with some studies reporting breast cancer in up to 25%–35% of female CNC patients (Kirschner et al., 2000; Rothenbuhler and Stratakis, 2010; Vaduva et al., 2024). Beyond breast cancer, PRKAR1A-associated Carney complex predisposes individuals to ovarian cancer, adrenocortical carcinoma, testicular tumors, gastric cancer, pancreatic neuroendocrine tumors, and pituitary adenomas, demonstrating the broad oncogenic consequences of PKA dysregulation (Kirschner et al., 2000; Bertherat et al., 2009; Rothenbuhler and Stratakis, 2010; Horvath et al., 2010; Vaduva et al., 2024). Although the PRKAR1A c.973+6T>C variant has not been previously reported in the hereditary cancer literature, its predicted splicing defect (exon skipping or nonsense-mediated decay) and dominant inheritance pattern are consistent with pathogenic mechanisms observed in other PRKAR1A mutations (Kirschner et al., 2000; Rothenbuhler and Stratakis, 2010; Horvath et al., 2010).

The identification of these variants has important implications for patient management and therapeutic strategy. Beginning with RAD50, individuals carrying mutations in this gene may benefit from enhanced surveillance protocols and from therapeutic strategies targeting DNA damage response pathways. The demonstration of PARP inhibitor efficacy in BRCA1/2-associated breast and ovarian cancers has opened new therapeutic avenues for patients with other homologous recombination (HR) deficiency-causing mutations. Current PARP inhibitors, including olaparib and rucaparib, have been approved by the FDA for treatment of BRCA1/2-positive breast and ovarian cancers, with olaparib specifically demonstrating durable response in platinum-sensitive ovarian carcinoma (Ledermann et al., 2014) and rucaparib showing efficacy in relapsed ovarian cancer (Swisher et al., 2017). These agents function by inhibiting poly(ADP-ribose) polymerase, preventing single-strand break repair and creating synthetic lethality in HR-deficient cells (Ledermann et al., 2014; Swisher et al., 2017). Given that RAD50 mutations similarly compromise the MRN complex and double-strand break repair, carriers of RAD50 mutations may be candidates for PARP inhibitor therapy, particularly in the context of breast and ovarian cancers. Additionally, traditional chemotherapy agents that induce DNA damage—such as platinum-based compounds (cisplatin, carboplatin) and topoisomerase inhibitors—may demonstrate enhanced efficacy in RAD50 carriers due to impaired DNA repair, warranting consideration in treatment selection and surveillance strategies.

For carriers of MSH4 loss-of-function mutations, therapeutic strategies must be tailored to the specific molecular phenotype of resulting tumors. While MSH4 is not part of the classical post-replication mismatch-repair machinery, its role in maintaining genomic integrity suggests that MSH4 carriers may develop tumors with elevated mutation burden and potential microsatellite instability (MSI). Recent advances in immunotherapy have demonstrated that MSI-high tumors respond robustly to immune checkpoint inhibitors (ICIs), including anti-PD-1 and anti-PD-L1 agents, due to their elevated neoantigen load (Kneitz et al., 2000; Wang et al., 1999; Cohen et al., 2006). Therefore, MSH4 mutation carriers who develop malignancies with evidence of MSI may be candidates for immunotherapy, whereas those with microsatellite-stable (MSS) tumors might benefit from traditional chemotherapy or targeted approaches. Comprehensive tumor profiling (including MSI/MSS status, tumor mutational burden, and immune checkpoint expression) would be essential for optimizing treatment selection in MSH4 carriers.

Moving to STAT6-driven malignancies, emerging therapeutic options offer promise for clinical application. STAT6-driven tumors, particularly those with dysregulated IL-4/IL-13 signaling, may respond to emerging targeted therapies. Several STAT6 inhibitors and IL-4/IL-13 pathway-targeting agents are currently under investigation in clinical trials (Pesu et al., 2008), with potential applications in STAT6-overexpressing breast cancers and other malignancies. Additionally, combination strategies pairing STAT6 inhibition with conventional chemotherapy or immunotherapy may enhance therapeutic efficacy by reducing metastatic potential and EMT-driven dissemination (Takeda and Akira, 2012; Ma et al., 2014; Park et al., 2013; Guo et al., 2018; Pesu et al., 2008). STAT6 carriers should undergo comprehensive tumor profiling to assess pathway activation status and inform treatment selection.

Finally, management of PRKAR1A variant carriers represents a unique challenge requiring multidisciplinary surveillance. PRKAR1A variant carriers, in the context of confirmed or suspected Carney complex, should undergo comprehensive surveillance for multiple tumor types including breast, ovarian, adrenocortical, testicular, gastric, and pancreatic malignancies (Kirschner et al., 2000; Bertherat et al., 2009; Rothenbuhler and Stratakis, 2010; Vaduva et al., 2024). Emerging therapies targeting PKA signaling represent potential future approaches, though these are not yet established in clinical practice (Kirschner et al., 2000; Bertherat et al., 2009; Rothenbuhler and Stratakis, 2010). Management of PRKAR1A carriers currently relies primarily on enhanced surveillance, genetic counseling of family members, and standard-of-care chemotherapy for malignancies that develop. Future clinical development of PKA inhibitors and other pathway-targeted approaches may expand therapeutic options for this population.

Beyond the clinical implications of these individual variants, our findings underscore broader issues regarding global representation in cancer genomics research. The Palestinian population represents an important resource for hereditary cancer research. Recent studies have focused on characterizing inherited cancer variants in Palestinian populations, contributing to our understanding of genetic predisposition in this region (Hamameh et al., 2017; Ghannam 2018; Ayesh et al., 2018). Consanguinity, a shared socio-cultural feature common across the Middle East and North Africa, influences genomic architecture and can increase the homozygosity of inherited variants, enriching both dominant and recessive pathogenic alleles and thereby enhancing the identifiability of disease-causing mutations (Qumseya et al., 2014; Hamameh et al., 2017). This characteristic makes Palestinian families valuable for mapping hereditary cancer variants that might be more difficult to identify in outbred populations. However, the limited availability of specialized genetic counseling services and the absence of population-specific germline variant databases underscore the urgent need for systematic genomic research in this region. Conducting research, recruiting families, and obtaining informed consent can be challenging due to restrictions on movement resulting from prevailing political conditions. Additionally, sociocultural factors—such as concerns about privacy, stigma associated with health conditions, and the influence of family-based decision-making—may reduce participation in genetic studies. Consequently, systematic genomic studies in this population remain limited, and knowledge of cancer-associated variant frequencies and clinical manifestations in Palestinian populations lags behind that of European and other well-studied populations.

Our findings demonstrate the potential of whole-exome sequencing to uncover clinically relevant variants and highlight the critical importance of developing a national genetic registry and variant database tailored to the Palestinian population. Establishing such infrastructure would facilitate more accurate variant interpretation, support early detection and risk stratification based on local allele frequencies, enable appropriate therapeutic selection informed by population-specific data, and ultimately advance precision oncology initiatives in the region.

While our study provides valuable initial insights, several limitations warrant acknowledgment. This study has several important limitations. The small number of families analyzed and the absence of large population-matched control cohorts limit the statistical power to establish population-specific allele frequencies and distinguish pathogenic variants from benign population variation. Although segregation analysis and *in silico* predictions support the potential pathogenicity of identified variants, functional studies in appropriate cellular and animal models are essential to establish their biological effects and cancer-predisposing mechanisms. Future research integrating transcriptomic, proteomic, and functional assays will be critical for validating the biological consequences of these variants. These limitations also inform important directions for future research. Larger population studies and the development of population-specific databases will improve statistical power to distinguish potential founder mutations from rare private variants. Given the potential for therapeutic targeting based on these variant types and the expanding landscape of precision oncology approaches, future studies should incorporate data on clinical outcomes, treatment responses, and long-term surveillance findings in carriers of these variants. Expanding genomic literacy among clinicians and integrating WES into diagnostic workflows could substantially enhance patient care, enable informed therapeutic decision-making, and improve genetic counseling across the Palestinian healthcare system.

## Data Availability

The original contributions presented in the study are publicly available. The WES data for this study is available at the NCBI database under the BioProject with the accession number PRJNA1030526.
